# Getting personal with collaborative sustainability experimentation: Reflections and recommendations from a transdisciplinary partnership with the Swedish craft beer sector

**DOI:** 10.1007/s13280-022-01751-x

**Published:** 2022-06-30

**Authors:** Barry Ness, Darin Wahl

**Affiliations:** 1 Lund University Centre for Sustainability Studies, LUCSUS, Lund University, Box 170, 22 100 Lund, Sweden; 2 Centre for Innovation Research, CIRCLE, Lund University, Lund, Sweden

**Keywords:** Collaborative, Craft beer, Small- and medium-sized enterprises (SMEs), Sustainability experimentation research, Transdisciplinarity, Urban living labs

## Abstract

This paper provides reflections on transdisciplinary knowledge coproduction and experimentation processes from sustainability researcher perspectives. It centers on a 5-year period of collaborative research with small- and medium-sized enterprises in an Urban Living Lab in the Swedish craft beer sector. Nine reflections cover a variety of issues and potentials encountered during numerous interactions with societal partners, and are structured by three levels: organizational, interpersonal and intrapersonal. Based on the reflections, authors then propose a set of seven considerations and recommendations for how to more effectively collaborate in such transdisciplinary constellations. The recommendations apply across the three levels, and describe an approach to collaborative research that asks the researcher to be open, transparent, self-aware and intentional, reflective and reflexive, and both adaptive and flexible. Furthermore, they aim to create soft structures to facilitate understanding and mutual learning, such as designating “organizational champions”, as well as to embed collaborative reflections into recurring meetings with partners to maintain trust and capture sustainability knock-on opportunities as they arise.

## Introduction

Research focused on promoting sustainability transitions via the employment of collaborative transdisciplinary approaches has rapidly expanded in recent years (Sengers et al. 2019; Lang and Wiek 2022; Tengö and Anderson 2022). It has grown into a diverse field, evolving from largely hypothetical contributions on how to coordinate and carry out concerted processes, including interaction of actors from in- and outside of academia, to concrete examinations of particular cases located around the globe (Lang et al. 2012; Darbellay 2015; Polk 2015; Zscheischler and Rogga 2015; Turnhout et al. 2019; Bergmann et al. 2021; Jahn et al. 2021). Methods have also advanced to focus on the process of knowledge co-production and co-design as well as the testing of sustainability solutions (Pohl and Hirsh-Hadorn 2007; 2008; Giuseppe 2015; Moser 2016; Felt et al. 2016; Renn 2021; Hakkarainen et al. 2021). This has also opened doors to the development of approaches and frameworks for the design, evaluation, and proper financing of more robust collaborative processes (Roux et al. 2010; Taylor et al. 2016; Luederitz et al. 2017; Bernert et al. forthcoming; Simon et al. 2018; Williams and Robinson 2020). The developments have also helped to better mainstream creative methodologies such as urban living labs (ULLs), Real-world Labs, and others (Schäpke et al. 2018; McCrory et al. 2020). These ‘lab’ approaches consist of academic and non-academics working in collaboration to conduct targeted sustainability-promoting experiments, in which actionable knowledge and innovative solutions are co-developed and trialed, with intentions to foster transdisciplinary learning and propel sustainable societal change (Roux et al. 2010; Bulkeley et al. 2016; Voytenko et al. 2016).

The increased focus on collaborative processes to promote sustainability transitions has also inspired targeted research on myriad fronts. Examples include a focus on the different actors that participate in the transdisciplinary process (Gaziulusoy et al. 2016; Simon et al. 2018), actor “convergence” processes (Daher et al. 2020), boundaries between scientific and social realms (Felt et al. 2016; Luederitz et al. 2021), and how learning takes place in collaborative processes (van Poeck et al. 2020). While the scholarship is making inroads into key principles for these processes (Fazey et al. 2018; Norström et al. 2020; Bergmann et al. 2021), it is less robust when it comes to the knowledge and skills needed to manage and drive transdisciplinary processes, particularly for one-on-one collaboration with actors outside of academic circles. In this paper, we reflect on the knowledge and experiences of academics engaged in collaboration to promote sustainability with the private sector, predominantly small- and medium-sized enterprises (SMEs) (Stubblefield-Loucks et al. 2010).

We provide reflections and insights from experiences working directly in transdisciplinary research, including motivations that foster robust knowledge co-creation between actors, and via the hands-on efforts to collaboratively explore and experiment with sustainable change. It adds to the growing body of literature that reflects-on and evaluates transdisciplinary processes (Roux et al. 2010; Cundill et al. 2015; Taylor et al. 2016; Luederitz et al. 2017; Bernert et al. forthcoming). However, here we attempt to get further “under the skin” and provide a detailed reflection of our engagement with societal actors via targeted insights that we have experienced over recent years.

This research centers on collaborative efforts from SustBeerLab, an ULL currently consisting of two targeted experiments to promote sustainability in the craft beverage production industry in southern Sweden. This paper is written from the perspective of university sustainability science researchers working over an extended period with a diversity of industry actors. The approach presented in this paper attempts to target the several strands of transdisciplinary research introduced above where transdisciplinary collaboration is foundational. In this reflection, we concentrate on two questions: *What are the significant possibilities and impediments when collaborating with actors external to academia to promote sustainable change*; and, *what measures can be taken to improve the collaborative processes?*

## Methods and reflection framework

This research takes a practice-based pragmatist approach to transdisciplinary sustainability interventions (Caniglia et al. 2020; Popa et al. 2015; West et al. 2019), focusing on how to engage with real-world problems, effectively and collaboratively. From this perspective, knowledge, action, and learning are iterative and cyclical, each informing the other. While the ULL is focused on interventions, we use ‘reflexivity as methodology’ to engage with evolving learning in ongoing experimentation (Beers and van Mierlo 2017; Fook 1999; Popa et al. 2015; West et al. 2019). Therefore, the progress of the ULL and experiments runs in parallel with inquiry into how that progress was achieved or impeded.

We use an *ex-post* approach to reflect on the collaborative process. Reflections were captured most often in conversation, both via email and voice (in-person and virtually), in the process of reviewing and updating progress on the experiments and recounting interactions with partners. We also carried out a series of reflection exercises and discussions based on our individual and joint interactions with the SMEs. The majority of transdisciplinary interactions were casual, unscheduled meetings, on-site without note-taking or recording. This was consciously chosen with researchers assuming the role of participant rather than observer. Systematic note-taking in this context was impractical, e.g., during the twice weekly visits to monitor the hops experiment.

These many interactions were not purposeful to collect insights on the process; instead, they were spontaneous interactions with light conversations, while the experiments were developed and maintained. However, these small interactions can be essential to trust building and establishing rapport with partners.

Targeted insights were synthesized and categorized in three reflection levels: organizational, interpersonal, and intrapersonal. While these levels are implicit in transdisciplinary research (cf. Lang et al. 2012; Wittmayer and Schäpke 2014; Bergmann et al. 2021), they are not explicitly discussed, and their interdependencies are even less explored. The organizational level encompasses the SME and lab structure and governance, in which the purpose and functioning of the lab are determined. The reflections for this level refer to discrepancies between how the lab is imagined to run at conception and how it actually progresses. The interpersonal level encompasses the space of interaction between lab actors. The intrapersonal level refers to the individuals themselves from an ‘I’ perspective. The interdependencies are many, but broadly we assume that an individual’s *inter alia* perceptions of self, personality, expectations, intentions, and emotions in general and in time impact the interactions they have with others. In turn these effect how they engaged with spaces in which they have influence, including priority-setting, decision-making, and time- and personnel-management.

We found that while separate levels were helpful for us to analyze, understand, and present our experiences, the levels also are deeply interdependent. The reflections evolved from several rounds of reflection and dialogue between the authors on the experiences and interactions in the lab process, as detailed above. These are presented as generalized insights in the reflection section below. We also offer a set of considerations and recommendations to improve up-close processes, each based on one or more reflections. They were likewise generated through a process of collaborative insight and reflection between authors. However, they are not structured around the three levels, as each apply across levels and covers multiple reflections.

### SustBeerLab project

SustBeerLab was established in 2017 in collaboration with craft beverage producers in southern Sweden. It emerged as an endorsed activity for Future Earth, an international network to advance research in support of transformations to sustainability. More recently, the lab has been a part of two university-led international research projects: Globally and Locally-sustainable Food-Water-Energy Innovation in Urban Living Labs (GLOCULL) and TRANSFORM. Each of the projects bring together an international consortium of ULLs. The former is linked through the broad theme of the food, water, and energy nexus; the latter has a focus on the sustainability transformation processes of SMEs. The broader objective of SustBeerLab is to increase the sustainability of the craft beverage sector through direct collaboration with regional beverage and ingredient producers. The collaborative research is carried out largely through planned and unplanned in-person and online meetings and especially, on-site interactions with partners while developing and conducting sustainability experimentation. These are bolstered by stakeholder workshops, researcher participation in craft alcohol producers’ association meetings (henceforth, ‘the association’) and regional craft beer festivals, partaking in panel discussions, individual brewery visits, thesis research (Carvalho et al. 2021), and ongoing social media engagement.

The core ULL team consists of three academic members, two of whom are researchers and the authors of this paper. A central figure of the ULL is the principal investigator and lead author of this paper. He is driven by an abiding personal interest in brewing beer, a passion to “tinker” with sustainability innovations, and to more deeply understand the methodology that joins the two. He is supported in the ULL by a PhD candidate and second author of this paper, who is a collaborator on all aspects of lab activities with research interests in collaborative learning and knowledge co-production and integration, particularly in processes of sustainable change. In addition, there are two main industry actors: one the head of the association, and the other, lead management for a partner brewery, each most-associated with experiments one and two, respectively (described below). Important to note that all but one core collaborator in the SustBeerLab identify as male, and all are from stable middle-class contexts. Therefore, the applicability of insights to more diverse contexts should be interrogated, adapted, or ignored as applies. At the brewery, there are several employees and the co-owners of the brewery with which we have regular interaction. One of the co-owners is quite active in the lab and experiment when technical and engineering expertise is needed. Since the experiment takes place on-site at the brewery, interactions with any number of these individuals can be expected on each visit, consisting of anything from a brief exchange of greetings to conversations on a wide variety of personal, brewing process, experiment, and/or sustainability related topics.

**Fig. 1:**
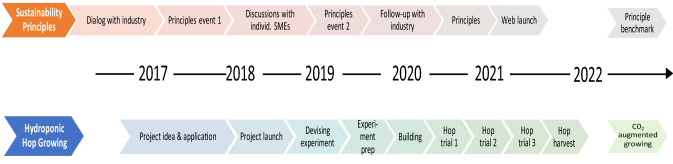
Main project phases of the 2 SustBeerLab experiments

A diversity of other industry actors (e.g., brewers, interest organization representatives, ingredient suppliers, and government officials) participate in lab activities in differing degrees, often by invitation to workshops (see sustainability principles below) or meetings. Meetings, especially for the hop growing, were not regular with non-core actors, instead they were often casual, as is the nature of this type of interactive research, focusing on building interest and knowledge sharing (e.g., greenhouse visits by other brewers). The funding for both projects was held by researchers, and, where applicable, allocated to non-academic actors to cover participation expenses. Figure 1 shows an event time line of each of the two main SustBeerLab experimentation initiatives.

### Sustainability principles

The initial targeted experiment was a set of co-created sustainability principles. This took place through two face-to-face workshops at Lund University initiated by the authors. To gain more robust perspectives, the participants included a diversity of industry actors and researchers from several different disciplinary backgrounds. Workshops entailed a presentation by the researchers, followed by discussion groups, each providing notes, which were collated into a master list of potential principles. A series of follow up meetings with individual brewers and industry representatives also took place to fine tune each principle. A follow up workshop was held for the same and other industry actors to discuss and provide written feedback on the principles. The feedback was synthesized and principles were finalized and made public by researchers in the spring 2021. Today, the principles are hosted by the association (www.skanesdryckesproducenter.com), and used as the basis for their sustainability policy, and other sustainability-related activities. Furthermore, the intention of the principles is to establish a set of region- and sector-relevant sustainability areas that producers can unite around and promote their sustainability work in their respective organizations.

### Hydroponic hops

The second experiment is the creation and testing of onsite beer ingredient production. It emerged as a new experiment as a part of the GLOCULL Project, and was carried out in collaboration with one regional craft brewery. The process also included other actors such as an architect/builder, and participation from several university researchers (e.g., agronomist), and research assistants depending on the experiment phase. The experiment aim is to establish and test a system where waste streams from brewing operations can be used to heat or cool a greenhouse attached to the brewery to grow hops hydroponically, year-round. The intention is for the hops then to be used for beer production as part of an integrated production system. Thus far, three trials have been completed with hydroponic and soil systems, examining different hop varieties, greenhouse temperatures, and plant light quantities. In upcoming trials, the intention is to augment growing rates with waste CO_2_ from the beer brewing process.

Experiment collaboration with the brewery has been ongoing with differing levels of intensity, for reasons detailed below. Greenhouse building commenced in March 2020 (coinciding with the onset of the COVID-19 pandemic in Europe), and the hop growing started in late-summer 2020. Construction of the greenhouse was largely carried out by researchers—with ongoing assistance from brewery employees and broad oversight from other actors (e.g., contractor, hydroponic system supplier, agronomists). The day-to-day growing has also been largely partaken by university researchers with assistance from brewery employees.

## Project, lab and experimentation reflections

### Organizational level

#### Experiment prioritization by actors

The broader challenges related to understanding and adapting to different organizational arrangements, cultures, and priorities, and unequal levels of commitment to the process by partners, has been highlighted in the transdisciplinarity literature (cf. Taylor et al. 2016; Simon et al. 2018; Bergmann et al. 2021). However, this literature seldom more deeply explores the frustrations and stress that can arise from the participatory process, and pathways to ameliorate them. Despite many sustainability scholars strongly prioritizing collaborative project work, the project likely will not be equally prioritized by collaborators, particularly SMEs. Although experimentation can help pave the way for new products and/or less-impactful ways of producing products, it likely will be viewed as ancillary. This is especially the case for SMEs where human resources are usually spread thin. Sustainability experimentation is not a core activity for most businesses, where priorities understandably are focused on value creation, revenue generation, troubleshooting, new product development, and business expansion. Augmenting this, scholars place significant priorities on producing research for peer-review publications, which in most cases is of marginal interest to SMEs.

These mis-matched priorities have, at times, created challenges for the hydroponic hop trials, which demanded close monitoring of indoor temperatures, water and nutrient levels, and lighting regimes. The on-site growing was meant to make monitoring relatively convenient, yet it was not always adequately attended to by the SME, especially when there were other priorities at the brewery. This led to several occasions of 45+°C temperatures in the greenhouse, or times when the hops went without water, thus having direct implications on growing rates, and ultimately the success of the trial.

#### Delays in the experimentation process

At a broader firm operations level, normal organizational schedules and activities can cause dependencies and delays in the experimentation processes. This aspect has been mentioned in other studies (Enengel et al. 2012; cf. Taylor et al. 2016; Bergmann et al 2021). For the hop growing, there were initial postponements caused by a physical brewery expansion process, which caused a temporary suspension in greenhouse planning and building. Furthermore, there were also modest interruptions created by employees taking leaves of absence (e.g., parental leave), having implications on both experiment operations and communication flows. Normally, such delays should be expected. However, the disturbances can lead to decreased time for actual experimentation, and foments anxiety for researchers on project progress reporting, deliverables fulfillment, funding allocation decisions, and researcher time allocation to the project. This can become a challenge, especially for projects with strict reporting and completion deadlines.

Similarly, there were delays in working with the association. This was due to the periodic and voluntary character of the group’s operations, and trying to fit our collaboration efforts into broader association agendas. This, for example, resulted in a rough year-long extension for the launch of principles.

#### Ebbs and flows of business production cycles

In addition to the above, the ebbs and flows of annual SME production cycles can greatly influence experiment participation. There are periods when operations are intense, and few human resources will be available for sustainability experimentation assistance. Furthermore, researchers must also be prepared to take advantage of situations when production periods wane, where extra efforts can be devoted to developing the experiment.

In our brewery collaboration, the spring represents busy months for beer production. It was also the time that greenhouse building was launched, leading to modest human resources availability by the brewery. Furthermore, December is a slow month for operations where strategic experiment planning and intensified work in the greenhouse could be carried out. Unfortunately, it also was the time in Sweden that that is least conducive for growing because of cold temperatures and poor ambient light.

#### Discrepancies between project demands and the co-creation process

Knowledge co-creation and integration, solution co-identification and testing, and reflexivity are cornerstones of transdisciplinary sustainability research (Lang et al. 2012; Popa et al. 2015; Taylor et al. 2016; Schäpke et al. 2018). However, as these processes are collaboratively defined during the project, it is difficult to foresee in the much earlier proposal-writing phase the actual direction the project will follow. This means that the project direction, as stated in the proposal, often is speculative (Rose and Maibaum 2020). In addition to this being a general hindrance to procuring project funding, this can also spark misunderstandings between project collaborators, as the specifics of project direction, project responsibilities, time and financial resource allocations have not been determined.

The hop growing experimentation option was co-created several months after the launch of the project, after numerous other experimentation options had been considered and discarded. However, the option also necessitated substantially different financial and human resource requirements than was written for in the strategically vague research application. Missing were the experimentation details and related allocations for greenhouse design, building permitting, and hop experiment monitoring. Many of these construction and maintenance responsibilities were not imagined by the project partners. This prompted the need for numerous conversations between partners regarding who has (or should have) responsibility for different aspects. As an outcome, partner time distributions did not correlate well with actual funding allocations.

### Interpersonal level

#### Maintaining good relationships in a dynamic environment

Establishing sustainable and lasting interpersonal relationships is commonplace in the transdisciplinary literature (Taylor et al. 2016; cf. Sellberg et al. 2021). Good working relationships come down to strong and positive interpersonal bonds between individuals. However, maintaining such relationships are rarely straightforward. Building trust and positive rapport with individual actors to facilitate healthy collaborative relationships, at a minimum for the duration of the project, is essential (Olsson et al. 2004; Harris and Lyon 2013; Bergmann et al. 2021). However, even if relationships are well-established, they are not maintained in a static collaborative environment. Instead, they are embedded in dynamic spaces where a variety of stressors, known and unknown, can create strained human interactions. Consequently, navigating the dynamic spaces of interpersonal relationships is imperative to the experimentation process. Shifting levels of commitment by key actors can have detrimental effects to the successful outcome of an experiment. The fluctuating levels of engagement can be due to myriad reasons, from commitments to the sustainability agenda, general job or personal stress, a concentration on accomplishing normal job responsibilities, to personality mismatches between individuals.

The expansion of the brewery and the installation of new brewing equipment, time demands for normal beer production, and brewing process troubleshooting, created situations where brewery staff did not always have the abilities or desires to assist with greenhouse building or with the maintenance of the experiments. This led to exchanges between particular project participants that often started as pleasant conversations, and then devolved into dank and awkward exchanges. Although tensions were never long-lasting, the situations sometime created an experimentation environment that ran counter to the intentions of transdisciplinary research. Augmenting the above, as hop growing got underway, so did the nature of the interpersonal exchanges. Discussions between researchers and SME leadership became quite technical and detail-specific (e.g., watering regimes, hop variety choice), while meetings discussing broader issues affecting and supporting the broader lab paled. Subsequently, a host of broader issues from perceived drops in interest and commitment, personality clashes, to greenhouse cost sharing, often weren’t discussed, fermenting frustrations for brewery management and researchers.

#### Sustainability knock-on effects

Despite the challenges described above, the interactions and relationship building between actors created a number of organizational sustainability knock-on effects*.* In our collaborations, especially with different craft brewers, discussions central to our research have led to action and/or conversations on alternative uses for spent grains, cooperative beer distribution and brewing supplies procurement, hot water recovery, profiling sustainability on SME websites, and additional augmentations of the hop growing system. This reflection links to findings by Luederitz and colleagues (2021) on how the interplay between planned (e.g., experiments) and emergent actions (e.g., discussions on new priorities) can spark new collective action (e.g., experiments). Without more objectively having examined this, the ongoing interactions presumably have led to much greater sustainability awareness among industry actors, and a better integration of sustainability thinking into business decisions.

#### Bridging scientific and societal project priorities

Scholarship on the transdisciplinary process have highlighted both the academic insights (e.g., methodological developments, study results) *and* societal (e.g., problem-solving) outcomes that emerge from the multi-actor process (cf. Lang et al. 2012; Taylor et al. 2016; Bergmann et al. 2021). However, our experiences have revealed that these processes are not always situated in separate realms. Close interactions can create circumstances where project participants from different facets of society have interests and stakes in aspects that fall outside of their traditional interest domains*.* More concretely, researchers can develop greater interest in advancing tangible societal solutions and developments, while non-academic actors can play a more central in the research process. This mashing of project roles can create processes where additional priorities, academic *and* societal, can be more effectively designed into, or evolve from, the experimentation process.

Our reflection is that our collaborations with nonacademic actors have crafted a growing curiosity and deeper comprehension and interest by them in the research process. Where SME employees were initially interested in maximizing hop yields, through adjusting growing parameters midcourse, researchers were focused on creating baseline growing systems and results where different parameters could be changed and studied in subsequent trials. Through ongoing dialog and collaboration, however, a greater understanding of, and interest in the research process quickly emerged by SME employees where more nuanced “scientific” discussions could take place about the hop growing.

Overall, what we have learned is that interpersonal relationships are very much dependent on the characteristics and habits of personalities that each individual has cultivated over their lives. The complexities of personalities often emerge as the collaboration intensifies. Therefore, the intrapersonal dimension (explored below) becomes an essential element for the transdisciplinary researcher; in other words, *who* and *how* we function as individuals matters for what we are able to accomplish as a team (Santoshi et al. 2010).

### Intrapersonal level

#### Researcher reflexivity

The organizational and interpersonal interactions during the experimentation opens the doors to reflections on how, we as researchers, see ourselves and act in the collaborative process. This self-examination not only prompts direct adaptation in how we interact in the transdisciplinary process, it also produces introspection on who we are as researchers. Reflexivity is critical for understanding interpersonal interactions (Wittmayer and Schäpke 2014; Popa et al. 2015; Fazey et al. 2018). Researchers engaged in transdisciplinary research are rarely just ‘doing science’, as all aspects of the transdisciplinary process are potentially important. The maintenance of ‘good’ trusting working relationships requires that most process interactions are reflected upon and interrogated. Such an awareness can help researchers to unpack why, as much possible, particular outcomes occurred. This goes beyond understanding motivations and priorities of others, as discussed above. Instead, it asks us to investigate our own behaviors and actions, both broadly and situationally, along with integrating our understanding of the influence of our positionality and personality. This is due, in part, to interactions in transdisciplinary contexts requiring the researcher to play many roles, often at the same time (Wittmayer and Schäpke 2014; Hilger et al. 2018). Our experiences throughout the process have shown that this can lead to internal conflict when, e.g., the sustainability normative position should be sidelined for easy, quick fixes, or to appease the local partner’s temperament at the time. These small moments, when misunderstood, can lead to resentment and frustration, outward or underlying, and were often the subject of many discussions between researchers where our own approaches were interrogated for word choice, tone, mode of communication, agenda, expectations, and intentions. It prompted questions such as what did we want/need from them, and them from us? Were we explicit and transparent; were they? Are we being overly demanding? What was my role during the interaction, and how did that contribute to outcomes?

#### Process patience

One of the largest skills learned by researchers from the experimentation process is patience (Höchtl et al. 2006; Grills 2020). The adage, “everything will take twice the amount of time than originally planned” for in the experimentation process is an understatement. In reality, the planning, purchase of materials, and building of the greenhouse took three- to four-times the time originally planned. This was often frustrating for researchers given a three-year duration of the GLOCULL research project. A several week delay with the building material delivery, on top of the building permitting process, the University’s procurement process, the absence of human resources to build the greenhouse, and a global pandemic were just a few of the many areas that caused a series of frustrations with researchers and nonacademic actors alike. The delays, in conjunction with periodic personality challenges, often necessitated much individual reflecting on actions and behaviors, and prompted exchanges between the research team to gain perspective on the experimentation process, and discussions on how to best keep it on track.

## Considerations and recommendations

We have formulated seven considerations and recommendations that address the above reflections. A summary of them can be found in Table 1 along with the association to each of the reflections.

**Table 1:** The table displays each of the seven considerations or recommendations and reflection that each corresponds to. The reflections are ranked by what we feel are their importance to the recommendation

Recommendations and considerations	Reflection section	Level relevance (organizational, interpersonal, intrapersonal)
Reflect on the influence of one's own positionality and personality	Researcher reflexivity; Maintaining good relationships in a dynamic environment; Process patience	Interpersonal, Intrapersonal
Develop skills and capacity to practice patience, compassion and empathy	Maintaining good relationships in a dynamic environment; Researcher reflexivity; Process patience; Delays in the experimentation process	Interpersonal, Intrapersonal
Learn the intentions & expectations of other particiapants	Experiment prioritization by actors; Delays in the experimentation process; Ebbs and flows of annual production cycles; Maintaining good relationships in a dynamic environment; Sustainability knock-on effects; Researcher reflexivity	Organizational, Interpersonal, Intrapersonal
Practice situational self-awareness and adaptability	Maintaining good relationships in a dynamic environment; Delays in the experimentation process; Experiment prioritization by actors; Researcher reflexivity	Organizational, Interpersonal, Intrapersonal
Integrate collaborative reflections to regular lab/experiment meetings	Experiment prioritization by actors; Delays in the experimentation process; Ebbs and flows of annual production cycles; Maintaining good relationships in a dynamic environment; Sustainability knock-on effects; Researcher reflexivity	Organizational, Interpersonal, Intrapersonal
Designate organizational champion(s)	Delays in the experimentation process; Experiment prioritization by actors; Ebbs and flows of annual production cycles; Maintaining good relationships in a dynamic environment; Sustainability knock-on effects; Researcher reflexivity	Organizational, Interpersonal, Intrapersonal
Strategically embed flexibility in proposal to anticipate change	Discrepancies between project demands and the co-creation process; Bridging scientific and societal project priorities; Delays in the experimentation process	Organizational, Interpersonal

### Positionality and self-reflection

Being an objective researcher who abides by established methods is a cornerstone of the scholarly profession. However, with transdisciplinary research it is important to enter into spaces with an openness to learn from and with others, and to have the skills and tools to help facilitate the creation of safe spaces to explore, learn, and co-create (Wamsler et al. 2020). Yet, outside of certain disciples where researchers are regularly embedded in social contexts (e.g., ethnography, cultural anthropology), academics are not always known for their social dexterity and the ability to backpocket their arrogances (cf. Cowan et al. 2019). Being aware of this generates the potential for the researcher to not only assess one’s own positionality, but also one’s mood and intentions ahead of interaction. More specifically, nurturing a tangible awareness of our personal actions, reactions, tendencies, and triggers are similarly important as the influences of our race, class, gender, and personal and societal privilege, which could be part of a combined positionality and personality awareness (Rose 1997; Moser 2008). Care, inclusivity, patience, self-awareness and reflexivity in the research process are a few leadership traits, especially for inter- and transdisciplinary researchers, that have only begun to be discussed and promoted (Cockburn et al. 2016; Knaggård et al. 2018; Kalman 2019; Fam et al. 2020; Care et al. 2021; Morss et al. 2021). *Researchers should therefore should not only consider their positionality in the transdisciplinary process; they should also reflect on their own personality characteristics and how these together can be displayed and perceived by others in multiple roles, situations, and conditions.*

### Inclusive, trusting collaborations

Related to the above, many research projects, collaborative projects included, can be dominated by scholars who control the experimentation process (Reich 2021). In the worst case, this can create a hierarchical structure where the nonacademic participants have little or no agency in the experimentation process. Researchers and nonacademic participants alike must actively work to foster flatter arrangements in the sustainability experimentation process (Care et al. 2021). As alternatives, Fam et al. (2020) describe a “We versus I” approach in collaborative, practice‐based research with a strong focus on learning instead of a concentration on individual achievement and project research objectives. Furthermore, Care et al. (2021) describe the interactions in greater detail via fostering new skills through self-organization, distributed responsibility and mutual respect. Alternatively, Basta et al. (2021), advocate the criteria of inclusiveness, equity, consistency, and flexibility when creating these more level constellations and building trusting connections within transdisciplinary research. Yet, to support these skills and criteria, we recommend that *researcher patience, compassion and empathy are practiced as important traits to create a process structure more conducive to inclusive, trusting collaborations*.

### Transparent expectations and intentions

The nature of communication in a transdisciplinary project is especially important to establish in the early phases of collaboration (Bergmann et al 2021). However, our experiences have shown that despite good intentions, the priorities, motivations and expectations of the various partners may go unexpressed. Therefore, we recommend that in order to set the proper foundation for collaborative experimentation, *each partner must take the time to learn the intentions and expectations of the others, and what each is capable and willing to devote to the partnership.*

Furthermore, getting used to working styles, levels of commitment, who has responsibility for what, are all aspects that must be worked out to avoid relationships going sour (Darabi et al 2021). Many questions could be discussed about the collaboration in advance (Bernert et al. forthcoming), for example: What are the purposes of the ULL and experiments; how are they connected with the interests and motivations of each partner; how are learning and reflexivity facilitated? It is also possible to dig into details: What sustainability impact will the experiment have; what will be assessed or monitored, and by whom? From this foundation, it can be possible to more easily navigate co-production paradoxes, such as the expressed versus actual project direction described above, since each partner is better aware of the expectations and intentions of the others. Moreover, when delays and other unforeseen obstacles do appear, the group will have the knowledge and structures in place (e.g., a dynamic understanding of communication and recurring meetings – see below) and the self- and group-awareness to adapt.

### Effective communication in dynamic settings

The experimentation literature recognizes the significance of active and open communication in collaborative research projects (cf. Wanner et al. 2018; Bergmann et al. 2021). However, we emphasize here that ‘good communication’ equates to more than frequent communication and exchange between participants. Communication is embedded in dynamic settings with joy, tension, and conflict. In addition to patience, empathy, and compassion stated above, *this necessitates the researcher to practice self-awareness and adaptability in order to navigate collaborative activities, maintain and further build trust among participants, and to foster positive, long-term relationships.* Instead, communicating effectively in dynamic settings integrates the intrapersonal with the interpersonal where participants attempt to understand, respond, and adapt to each other. This may require changes in the how, where, or frequency of communication, including addressing difficult or uncomfortable topics to prevent a fermenting of negativity.

### Regular collaborative reflections

Depending on the sustainability experiment and the level of collaboration, regular, scheduled meetings between partners must take place during the experimentation process (Bergmann et al. 2021). These regular recurring meetings can be useful in discussing a wide variety of lab and experiment activities and issues including daily tasks, responsibilities, delays, or upcoming production ebbs and flows.

However, the regular meetings still may not be sufficient to continue to build or maintain trusting relationships, especially during periods of organizational tension. Discovering where interpersonal disagreements and frustrations originate from is key to maintaining the trust space discussed above. However, individuals are not always upfront, or even aware of the real reasons for their emotions or thoughts, especially as they arise. Adding to the complication, we most often rely on our own personalities and experiences to navigate the disagreement, unaware that the frustrations and inconsistencies may be our own. Therefore, for encounters to be as effective, responsive, and relevant as possible, *we recommend to add collaborative reflection to assess the progress of the experiment, concentrating on the actions, ideas, expectations, and/or obstacles that are contributing to success, delay or other hindrances.* These discussions can increase the transparency of agendas, and create spaces to practice transdisciplinary dialogue that can lead to mutual learning and knowledge integration that is central to co-producing new, relevant, and actionable knowledge (Moreno-Cely et al. 2021; Dalla-Fontana et al. 2021).

Moreover, through the regular reflections outlined above, it is possible to recognize emergent sustainability related outcomes as they develop. These often-unwritten sustainability knock-on effects that come for the experimentation should not be underestimated. Beyond the much focused-on co-learning and capacity building outcomes (Kläy et al. 2015; Schäpke et al. 2018; cf. Fam et al. 2020), other important outcomes such as facilitating advances in sustainability thinking or expanded implementation ideas must also be viewed as essential in the process. If done right, it is these characteristics that can propel the project into new experimentation phases and help strengthen pro-sustainability actions in the SME and other organizations.

### Organizational champions

Proper communication flows to navigate aspects such as production ebbs and flows and ensure the proper prioritization of the ULL and/or experiment are essential for success. To help accomplish this, the academic literature has emphasized the importance of a leader, intermediary, or a boundary spanner (e.g., a “champion”) to drive and mediate the sustainability experimentation or transition process (Cash et al. 2003; Wiesner et al. 2018; Kivimaa et al. 2019; Kundurpi et al. 2021). We agree that a designated individual, often an academic, that has responsibility for oversight of the project is essential for the smooth operation of the experiment. However, our experiences also have demonstrated that it is not always sufficient to have a single individual to effectively drive the process. Therefore, *we recommend to designate “organizational champions” to help ensure the facilitation and smooth running of the project*. This individual/s act as the main organizational contact person(s), as well as the driver(s) of the experimentation activities within the organization. In most cases, they work on-site at the location of the experiment. They may be in addition to an intermediary, who has broader project responsibilities (Kivimaa et al. 2019). We also suggest, to help ensure that the champions are sufficiently devoted to experimentation activities, that a percentage of their time should be set-aside and compensated for. In addition to main experimentation responsibilities, they will also work to inform and motivate others about the experiment and its context. Furthermore, the organizational champion/s can act as the contact person for all issues about the experiment process, including the daily maintenance routines, personality conflicts, and project outreach efforts.

### Time planning

Project durations can greatly vary. However, as presented above, our experiences with projects with experimentation is that that they take much more time than originally planned for due to the delay factors. Furthermore, as ULLs are carried out in societal spaces, the researcher should expect that perturbations will occur in the process. In such cases, the process may require pivots in the experimentation planning and execution process where outcomes and outputs are adjusted accordingly. It is imperative that ambitions of the sustainability experiment be adjusted according to the length of the project with ample amounts of empty time to account for delays, mistakes or experimentation failure. This also means that it can *be strategic in the application writing phase of the project to include flexibility in the outcomes and outputs to anticipate change as the project progresses.* Again, we reiterate that this requires process patience, even though the experiment has not reached the stated outcomes, or that reporting deadlines are near.

## Final remarks

In this paper, we have provided nine reflections on a sustainability knowledge co-production and experimentation process from a sustainability researcher perspective. We structured these reflections around three levels: organizational, inter-, and intrapersonal, and focused significantly on collaboration with SMEs. These levels proved to be critical in interrogating the issues we were facing during the progress of the lab. Without the levels, there was not a clear way to separate out reflexive spaces in which we could see what we ourselves brought to a situation that could have influenced an outcome. In other words, it forced us to consider ways in which our own ways of engagement could be problem-causing, and then follow that into interactions with others, and the governance of lab activities. The recommendations, however, did not fit into these levels easily, due to their interdependence. Therefore, the levels served best as an analytical guide.

Based on these reflections, we have developed a set of seven considerations and recommendations for how to interact more effectively in these processes. Our intentions have been to derive a number of insights that both enhance other ongoing research in the field, as well as deepen comprehensions of transdisciplinary processes, especially as they pertain to collaboration. As a result of this reflective process, we have learned that a willingness to learn to collaborate and co-create with others goes hand in hand with the need to reflect on oneself and what we, as researchers, bring to the partnerships beyond mere expertise in science.

Finally, many of the suggestions advocated in this paper are those that can be taken by sustainability researchers to improve the transdisciplinary process. However, there is still a host of broader structural changes that are still needed in order for this form of research to thrive. How we finance sustainability research, how universities are structured, what denotes project success, and who is able to partake in this research are just a sampling of issues that need to be discussed and acted upon in parallel with the evolution of the approaches.
